# Diabetes in women and health-related quality of life in the whole family: a structural equation modeling

**DOI:** 10.1186/s12955-019-1252-4

**Published:** 2019-12-05

**Authors:** Mina Moeineslam, Parisa Amiri, Mehrdad Karimi, Sara Jalali-Farahani, Niloofar Shiva, Fereidoun Azizi

**Affiliations:** 1 Research Center for Social Determinants of Health, Research Institute for Endocrine Sciences, Shahid Beheshti University of Medical Sciences, Tehran, Iran; 2 Department of Epidemiology and Biostatistics, School of Public Health, Tehran University of Medical Sciences, Tehran, Iran; 3 Endocrine Research Center, Research Institute for Endocrine Sciences, Shahid Beheshti University of Medical Sciences, Tehran, Iran

**Keywords:** Type 2 diabetes, Health-related quality of life, Women, Family members, Structural equation modeling

## Abstract

**Background:**

Although **s**everal studies indicate the effects of diabetes type 2 on health-related quality of life (HRQoL) in female subjects, the related impact of the disease on HRQoL in their family members has rarely been the focus of the empirical research. In this study we aim to investigate associations between diabetes in women and the HRQoL in these women and their family members, using the structural equation modeling (SEM).

**Methods:**

This family-based study was conducted on 794 women (11.1% with diabetes) as well as their spouses and children who participated in the Tehran Lipid and Glucose Study (TLGS) from 2014 to 2016. Data on HRQoL were collected using the Iranian version of the Short-Form 12-Item Health Survey version 2 (SF-12v2) and the Pediatric Quality of Life Inventory version™ 4.0 (PedsQL). SEM was conducted to evaluate the network of associations among studied variables. Data were analyzed using IBM SPSS Statistics & AMOS version 23 software.

**Results:**

Mean age of women was 41.37 ± 5.32 years. Diabetes in women significantly affected their mental HRQoL (β = − 0.11, *P* < 0.01) but showed no significant direct associations with physical and mental HRQoL in their spouses or their children. However, poor mental HRQoL in women with diabetes was associated with decrease in both physical (β = − 0.02, *P* = 0.013) and mental (β = − 0.03, *P* < 0.01) HRQoL in their spouses and total HRQoL score in children (β = − 0.02, *P* < 0.01).

**Conclusions:**

Among women with diabetes type 2, beyond its effect on their mental HRQoL per se, demonstrated a negative association with the self-assessment of health status in their spouses and children. Such familial consequences are mainly attributed to the negative effect of the disease on the mental rather than the physical HRQoL in women with diabetes.

## Introduction

Diabetes is a fast emerging public health problem and the fourth leading cause of disability, worldwide [1]. It is estimated that 425 million adults globally suffer from diabetes, of which 203.9 million are women [2]. Across IDF regions, the Middle East and North Africa (MENA) region has the second highest rate of diabetes [2]. Type 2 diabetes is the most prevalent type of diabetes, including 90 to 95% of patients [3]. In Iran, epidemiological studies show that the prevalence of this disease increases annually around 0.4% in the general population, aged> 20 years, being 1.7% higher in women than in men [4]. Beyond physical complications, diabetes can lead to several psycho-behavioral problems such as depression [5], anxiety [6] and poor lifestyle behaviors [7] which ultimately results in decline in their health-related quality of life (HRQoL).

Health-related quality of life is a broad and multidimensional concept that subjectively evaluates the physical, psychological, and social health status of individuals and is influenced by their understanding, experiences and expectations [8]. Systematic reviews conducted on studies of different countries and cultures [9, 10] as well as Iran [11], indicate that diabetes can affect all aspects of HRQoL. Compared to general populations, individuals with diabetes, especially women, are more likely to be inactive and overweight and experience more negative emotional states which can adversely affect their HRQoL [8, 12]. Existing evidence shows that women have greater concern about diabetes and lower adaptability to this metabolic disorder [13]. Since, women prefer to identify themselves with the role of care provider in the family and are less willing to accept support from other members of family [14], managing illness and adherence to diet and exercise is more difficult for them, with most reporting higher degrees of distress [15].

Although the effects of diabetes on the patients’ HRQoL are well-documented [9, 11], data from studies on the familial effects of this disease are limited. Family members of a person with diabetes have many concerns about the complications of disease, limited social interactions and they feel insecure about the future [16]. Living with a person with diabetes, may lead to negligence in the needs and demands of the other family members [17] and decreased familial well-being [18, 19]. Considering the different roles of women in their families, their diabetes may have different effects on family members. In this regard findings of a study showed when a mother suffers from a chronic illness like diabetes, it may reduce the quality of mother-child interactions and parenting behaviors which adversely affect the HRQoL of their children [20]. However, to the best of our knowledge there is no study documented on the effects of women’s diabetes on the HRQoL of their husbands, specifically.

Hence, to understand the burden of type 2 diabetes in societies, considering the complications of this disease beyond its individual effects seems vital. Although in recent decades, Iranian women have higher levels of education and can manage social responsibilities, their caring role in the demands of their families ranks first [21]. Similar to many other countries, Iranian women are more likely to report poor HRQoL than men [11]. This issue, along with the transition mentioned in our society, highlights the importance of considering the effects of chronic diseases, including diabetes type 2, on the health status of all family members. The current study, using the structural equation modeling (SEM) aimed to evaluate the path via which diabetes in women influences the HRQoL of the whole family.

## Methods

This study conducted within the framework of the Tehran Lipid and Glucose Study (TLGS), seeks to investigate and monitor the main cardiovascular risk factors in a representative population recruited from among residents of district 13 of Tehran, the capital of Iran. The TLGS aims at changing and improving lifestyles and preventing non-communicable diseases. The TLGS is divided into two phases, a cross-sectional and a prospective ongoing follow-up study, designed to continue collecting data for at least 20 years with assessments at 3-year intervals. Details of the rationale and design of the TLGS have been published elsewhere [22, 23]. For the current analysis, data of 794 married women who participated in the TLGS during 2014–2016, who had at least one school-aged child and had complete data on diabetes, socio-demographics, BMI and HRQoL were analyzed. A written informed consent form was completed and signed by all participants. This study was approved by the ethics committee of Research Institute of Endocrine Sciences, Shahid Beheshti University of Medical Sciences, Tehran, Iran.

### Measurements

Trained interviewers collected socio-demographic data, including age, education level and employment status and anthropometric data, including weight and height of adult participants, as well as age and gender of children. To obtain HRQoL information, all participants (adults and children) were interviewed using (i) the Short-Form 12-Item Health Survey version 2 (SF-12v2) and (ii) the Pediatric Quality of Life Inventory version™ 4.0 (PedsQL) respectively.

(i) *SF-12v2:* This questionnaire is the short form of Health Survey SF-36 and was developed to measure eight domains of physical and mental health. The subscales of Physical Component Summary (PCS-12) are General Health (GH), Physical Functioning (PF), Role Physical (RP), and Body Pain (BP); subscales of Mental Component Summary (MCS-12) are Vitality (VT), Social Functioning (SF), Role Emotional (RE), and Mental Health (MH). Each health domain score ranged from 0 to 100, with higher scores indicating better health [24]. Good reliability and validity of the Iranian version of this questionnaire have been reported previously; cronbach’s alpha was 0.87 for the PCS-12 and 0.82 for the MCS-12 [25].

(ii) *PedsQL™ 4.0:* This 23-item questionnaire includes the child self-report and the parent-proxy report, and has four subscales: Physical Functioning, Emotional Functioning, Social Functioning and School Functioning. In this 5-point response scale for ease of interpretability, items are transformed to a 0 to 100 scale (0 = 100, 1 = 75, 2 = 50, 3 = 25, 4 = 0) with a higher score indicating better HRQoL. The validity and reliability of this questionnaire used for Iranian children [26] and adolescents [27] have been previously confirmed.

### Definitions

#### Diabetes type 2

According to the guidelines of the American Diabetes Association, diabetes type 2 was defined as FPG ≥126 mg/dl, or 2-h PCPG ≥11.1 mg/dl or taking anti-diabetic medication [28].

#### Obesity

In the current study, obesity is defined as BMI ≥ 30.0 kg/m^2^.

### Statistical analysis

Mean ± SD for continuous variables and frequency (%) for categorical ones are reported as descriptive statistics. Independent samples t-test and chi-square test were conducted to compare mean and distribution of variables across women’s diabetes status, respectively. To evaluate the inter-relationships among study variables, we used Structural Equations Modeling (SEM). SEM is a statistical technique that fits networks of constructs to data and simultaneous associations among variables. The conceptual framework of the relationships was hypothesized by the researcher in the SEM. To evaluate the appropriateness of hypothesized models the fit indices of SEM and their acceptable threshold levels are reported [29].

**Fig. 1:**
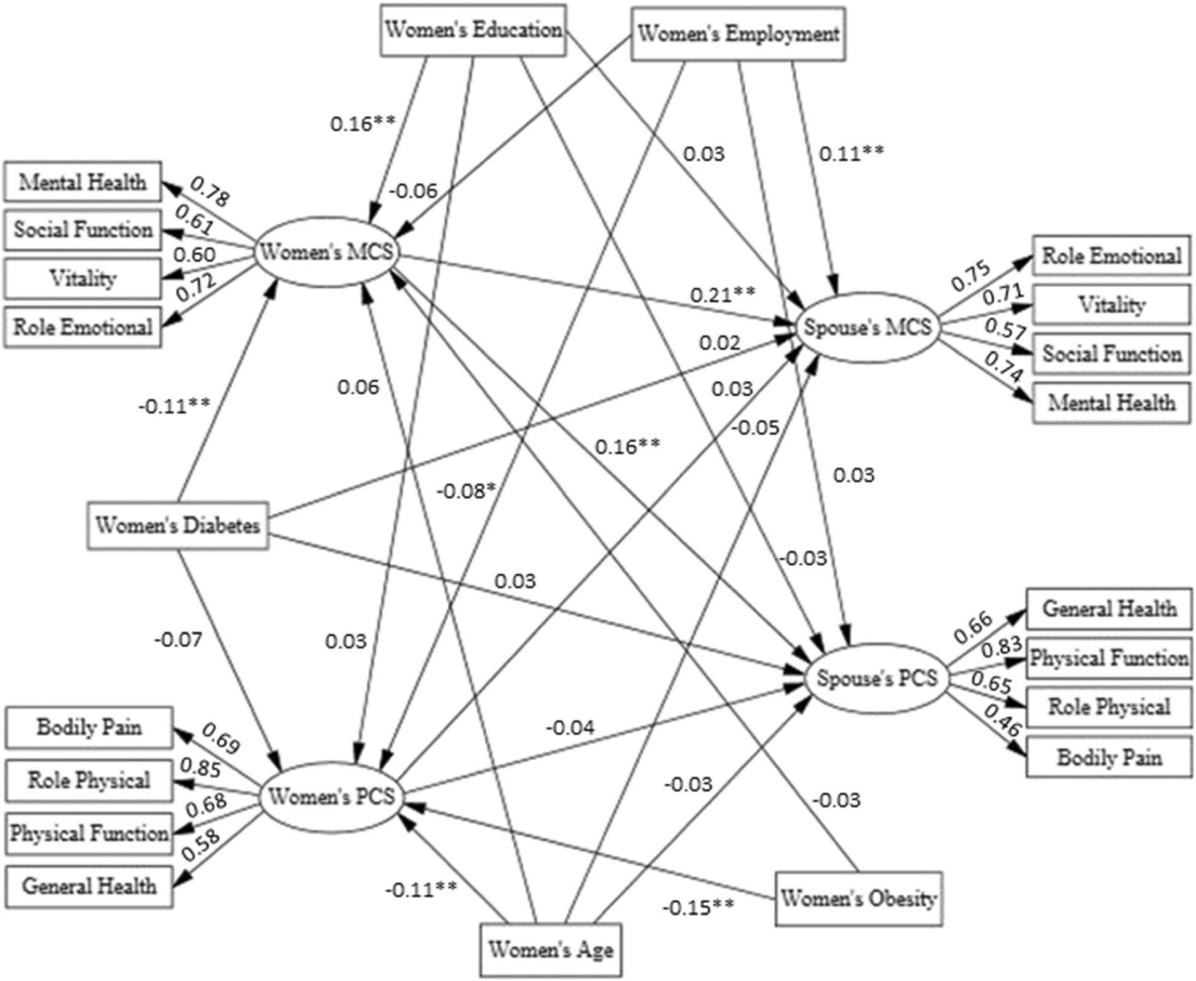
Structural model 1: Diabetes in women and their HRQoL and their spouses considering influential variables. Standardized estimates are illustrated above pathways. * *P* < 0.05, ** *P* < 0.01. All factor loadings in measurement model of latent variables were significant (*P* < 0.001). Model fit indices including Chi-Square = 416.72, Degrees of Freedom (DF) = 150, Chi-Square/DF = 2.78, Standardized Root Mean Square Residual (SRMR) = 0.039, Root Mean Square Error of Approximation (RMSEA) = 0.047, Comparative Fit Index (CFI) = 0.94, Goodness of Fit Index (GFI) = 0.95, Adjusted Goodness of Fit Index (AGFI) = 0.93, Normed Fit Index (NFI) = 0.91 and Incremental Fit Index (IFI) = 0.94 display acceptable thresholds and confirm the model appropriateness

**Fig. 2:**
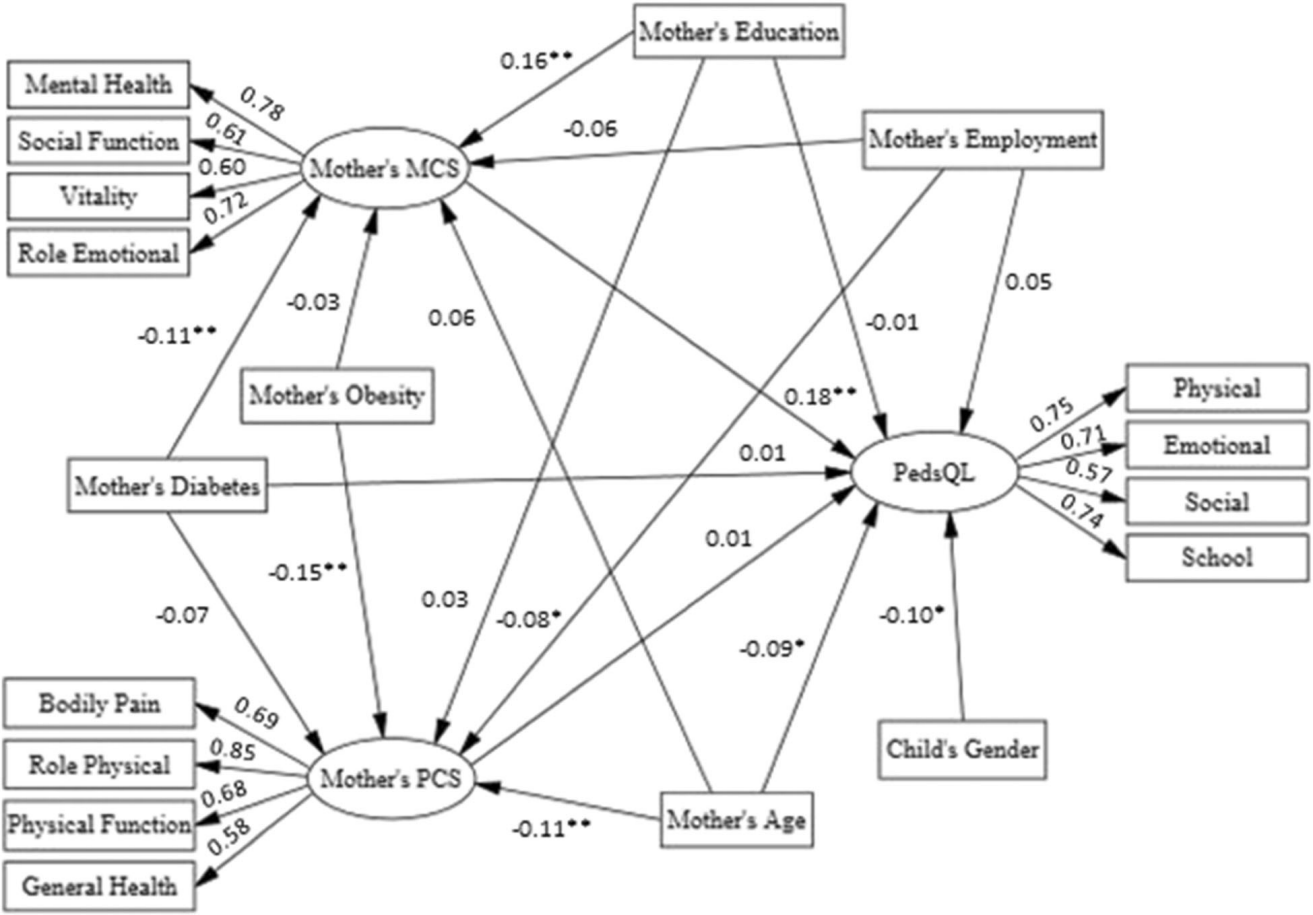
Structural model 2: Diabetes in women and their HRQoL and their children considering influential variables. Standardized estimates are illustrated above pathways. * P < 0.05, ** P < 0.01. All factor loadings in measurement model of latent variables were significant (P < 0.001). Model fit indices including Chi-Square = 325.10, DF = 106, Chi-Square/DF = 3.06, SRMR = 0.048, RMSEA = 0.051, CFI = 0.93, GFI = 0.96, AGFI = 0.94, NFI = 0.90 and IFI = 0.93 display acceptable thresholds and confirm the model appropriateness

In this study the hypothesized conceptual models of the association among women’s diabetes and family members quality of life mediated by women’s quality of life are shown in Figs. 1 and 2. In the tested SEM models, women’s diabetes status (no, yes) was considered as the observed exogenous variable, child’s and spouse’s quality of life were endogenous variables and women’s quality of life was considered to be the mediator. Familial quality of life scales were the latent constructs and their subscales were considered the observed indicators. Previous findings have indicated significant associations between socio-demographic factors and HRQoL [30]; hence, in the current analysis, socio-demographic factors, including women’s age, education and employment status and child’s gender were considered as potential confounders and entered in the model. Maximum likelihood was used as the estimation method and 95% bootstrap confidence intervals were reported for estimated parameters. IBM SPSS Statistics & AMOS version 23 were used for statistical analysis and structural modeling.

## Results

The descriptive statistics of demographic variables for women, their spouses and children have been shown in Table 1. Mean ages of women and their spouses were 41.37 ± 5.32 and 47.24 ± 5.83 years, respectively. Mean ages of children (51.3% boys) were 13.44 ± 3.10 and 13.45 ± 3.2 years in boys and girls respectively. In total 11.1% of women had diabetes and mean ages of women with and without diabetes were 43.87 ± 5.79 and 41.06 ± 5.17 years, respectively (*P* < 0.001). Educational status in women with and without diabetes differed significantly (*P* = 0.01). More percentage of women without diabetes had higher levels of education, compared to those with diabetes. Women without diabetes were more likely to be non-obese (*P* < 0.01). The mean ages for spouses of women with and without diabetes were 49.29 ± 6.60 and 46.96 ± 5.68, respectively (*P* < 0.01). Spouse’s education, employment status as well as child’s age and sex did not differ significantly between women with and without diabetes.

**Table 1:** Characteristics of women with and without diabetes, their spouses and children

Variables	Total (*n* = 794)	Without diabetes (*n* = 706)	With diabetes (*n* = 88)	*P*-value
Women’s Variables				
Age (y)	41.37 ± 5.32	41.06 ± 5.17	43.87 ± 5.79	< 0.001
Education				0.01
Primary School	108 (13.6)	95 (13.5)	13 (14.8)	
Secondary School	444 (55.9)	384 (54.4)	60 (68.2)	
Higher	242 (30.5)	227 (32.2)	15 (17.0)	
Employment				0.13
Housewife	638 (80.4)	562 (79.6)	76 (86.4)	
Employed	156 (19.6)	144 (20.4)	12 (13.6)	
Weight Status				< 0.01
Obese	265 (33.4)	221 (31.3)	44 (50.0)	
Non-obese	529 (66.6)	485 (68.7)	44 (50.0)	
Spouse’s Variables				
Age (yr)	47.24 ± 5.83	46.96 ± 5.68	49.29 ± 6.60	< 0.01
Education				0.48
Primary School	123 (19.1)	106 (18.5)	17 (23.86)	
Secondary School	320 (49.7)	284 (49.7)	36 (50.0)	
Higher	201 (31.2)	182 (31.8)	19 (26.4)	
Employment				0.07
Employed	606 (94.0)	542 (94.6)	64 (88.9)	
Unemployed	39 (6.0)	31 (5.4)	8 (11.1)	
Child’s Variables				
Age (yr)	13.44 ± 3.10	13.38 ± 3.09	13.93 ± 3.07	0.11
Sex				1.00
Boy	407 (51.3)	362 (51.3)	45 (51.1)	
Girl	387 (48.7)	344 (48.7)	43 (48.9)	

Data are represented as Mean ± SD or frequency (percent)

The descriptive statistics of HRQoL scores in women, spouses and children are presented in Table 2. Women with diabetes had significantly lower scores than women without diabetes in RP (67.3 ± 23.1 vs 73.5 ± 22.5 respectively, *P* = 0.01), PF (74.6 ± 25.9 vs. 80.8 ± 25.04 respectively, *P* < 0.01) and GH (46.85 ± 22.6 vs. 51.6 ± 21.5 respectively, *P* = 0.01). Compared to their counterparts without diabetes, women with diabetes also had significantly lower SF (65.1 ± 31.95 vs. 76.94 ± 27.1 respectively, *P* < 0.01) and VT (52.7 ± 25.1 vs. 60.7 ± 23.5 respectively, *P* < 0.01). Similarly, the PCS (44.5 ± 9.7 vs. 47.1 ± 8.2, *P* < 0.01) and MCS (43.7 ± 11.2 vs. 46.7 ± 10.4, *P* = 0.02) scores differed significantly between women with and without diabetes, respectively. There were no significant differences between HRQoL scores for spouses of women with and without diabetes, except for PF (85.2 ± 20.8 vs. 89.1 ± 19.3 respectively, *P* = 0.01). Also, based on child self-reports, total and subscales scores of HRQoL did not differ significantly between children of mothers with and without diabetes.

**Table 2:** Health-related quality of life in women with and without diabetes, their spouses and children

Variables	Total (*n* = 794)	Without diabetes (*n* = 706)	With diabetes (*n* = 88)	*P*-value
Women				
PCS	46.83 ± 8.38	47.12 ± 8.17	44.49 ± 9.66	< 0.01
Bodily Pain	72.04 ± 23.67	72.53 ± 23.28	68.10 ± 26.40	0.24
Role Physical	72.80 ± 22.65	73.48 ± 22.51	67.28 ± 23.08	0.01
Physical Functioning	80.07 ± 25.19	80.76 ± 25.04	74.56 ± 25.89	< 0.01
General Health	51.07 ± 21.66	51.60 ± 21.49	46.85 ± 22.60	0.01
MCS	46.35 ± 10.48	46.68 ± 10.35	43.74 ± 11.19	0.02
Mental Health	66.65 ± 20.43	67.06 ± 20.30	63.32 ± 21.27	0.09
Social Functioning	75.63 ± 27.86	76.94 ± 27.05	65.14 ± 31.95	< 0.01
Vitality	59.79 ± 23.80	60.68 ± 23.49	52.70 ± 25.14	< 0.01
Role Emotional	68.97 ± 24.34	69.57 ± 24.05	64.15 ± 26.19	0.05
Spouses				
PCS	49.84 ± 6.84	49.84 ± 6.78	49.85 ± 7.31	0.80
Bodily Pain	83.16 ± 19.82	83.04 ± 19.89	84.15 ± 19.35	0.64
Role Physical	85.18 ± 17.73	84.99 ± 17.94	86.73 ± 15.90	0.63
Physical Functioning	86.69 ± 19.49	89.13 ± 19.29	85.22 ± 20.80	0.01
General Health	53.97 ± 20.23	53.70 ± 20.09	56.16 ± 21.37	0.16
MCS	50.26 ± 9.72	50.25 ± 9.65	50.31 ± 10.18	0.77
Mental Health	72.84 ± 18.84	72.82 ± 19.06	73.05 ± 17.12	0.87
Social Functioning	82.88 ± 22.65	83.02 ± 22.87	81.68 ± 20.97	0.26
Vitality	69.60 ± 22.78	69.79 ± 22.50	68.04 ± 25.03	0.67
Role Emotional	80.02 ± 20.59	80.16 ± 20.45	78.92 ± 21.80	0.33
Children				
PedsQL	85.05 ± 10.66	85.13 ± 10.85	84.44 ± 9.06	0.21
Physical	89.85 ± 10.85	89.82 ± 11.04	90.06 ± 8.91	0.50
Emotional	74.04 ± 18.33	74.12 ± 18.47	73.41 ± 17.25	0.60
Social	88.51 ± 13.52	88.67 ± 13.43	87.27 ± 14.20	0.44
School	84.92 ± 13.60	85.08 ± 13.72	83.64 ± 12.54	0.13

Data are represented as Mean ± SD

The standardized estimation of associations among diabetes and HRQoL in women with HRQoL of their spouses and children are illustrated in Figs. 1 and 2. Fit indices, below the figures, indicate good and acceptable thresholds for SEMs in the evaluation of hypothesized conceptual frameworks. According to both structural models (Table 3), higher age (β = − 0.11, *P* < 0.01) and obesity (β = − 0.15, *P* < 0.001) decreased women’s PCS. Higher education was positively (β = 0.16, *P* < 0.001) and diabetes was negatively (β = − 0.11, *P* < 0.01) associated with women’s MCS.

**Table 3:** Standardized coefficients (95% bootstrap-CI) of the structural model used for examining women’s diabetes status and their HRQoL related to child’s PedsQL

Predictors	Dependent	β (95% CI)	P-value
Structural Model 1			
Women’s Age	Women’s PCS	−0.11 (−0.18, −0.03)	< 0.01
Education		0.03 (−0.05, 0.11)	0.41
Employment		− 0.08 (− 0.15, − 0.01)	0.04
Obesity		− 0.15 (− 0.23, − 0.07)	< 0.001
Diabetes		− 0.07 (− 0.15, 0.01)	0.08
Women’s Age	Women’s MCS	0.06 (− 0.02, 0.14)	0.14
Education		0.16 (0.08, 0.24)	< 0.001
Employment		−0.06 (− 0.14, 0.02)	0.13
Obesity		−0.03 (− 0.12, 0.05)	0.44
Diabetes		−0.11 (− 0.19, − 0.03)	< 0.01
Women’s Age	Spouse’s PCS	− 0.03 (− 0.12, 0.06)	0.50
Education		−0.03 (− 0.11, 0.05)	0.47
Employment		0.03 (−0.04, 0.10)	0.47
Diabetes		0.03 (−0.05, 0.11)	0.46
PCS		−0.04 (− 0.16, 0.08)	0.53
MCS		0.16 (0.04, 0.29)	0.01
Women’s Age	Spouse’s MCS	−0.05 (− 0.13, 0.03)	0.27
Education		0.03 (−0.06, 0.12)	0.47
Employment		0.11 (0.03, 0.19)	< 0.01
Diabetes		0.02 (−0.06, 0.09)	0.69
PCS		0.03 (−0.13, 0.18)	0.73
MCS		0.21 (0.07, 0.36)	< 0.01
Structural Model 2			
Mother’s Age	PedsQL	−0.09 (−0.17, − 0.01)	0.04
Education		−0.01 (− 0.11, 0.08)	0.74
Employment		0.05 (−0.04, 0.14)	0.27
Diabetes		0.01 (−0.06, 0.09)	0.72
PCS		0.01 (−0.12, 0.13)	0.96
MCS		0.18 (0.04, 0.31)	0.01
Child’s gender		−0.10 (−0.18, − 0.02)	0.02

Correlation between Maternal MCS and PCS = 0.63, *P* < 0.001

Correlation between Maternal age and education = − 0.12, *P* < 0.001

Correlation between Maternal education and employment = − 0.37, *P* < 0.001

Correlation between Maternal obesity and diabetes = 0.12, *P* < 0.001

Correlation between spouse’s MCS and PCS = 0.49, *P* < 0.001

Table 3 (Model 1) illustrates the associations of diabetes in women with the HRQoL of their spouses. Diabetes in women did not have a significant direct effect on their spouse’s PCS (β = 0.03, *P* = 0.46) and MCS (β = 0.02, *P* = 0.69). Furthermore, women’s PCS did not have significant effects on spouse’s MCS and PCS, whereas women’s MCS was found to have direct associations with their spouse’s PCS (β = 0.16, *P* = 0.01) and MCS (β = 0.21, *P* < 0.01). As indicated in Fig. 1, women’s diabetes indirectly reduced spouse’s PCS (β = − 0.02; 95%CI = (− 0.04 to − 0.01), *P* = 0.013) and MCS (β = − 0.03; 95%CI = (− 0.05 to − 0.01), *P* < 0.01) via women’s MCS. Regarding demographic variables, none of these characteristics in women were associated with their spouse’s PCS, whereas employment in women was directly associated with their spouse’s MCS (β = 0.11, *P* < 0.01). Although, educational level in women did not have a direct effect on their spouse’s HRQoL, it indirectly increased their spouse’s PCS (β = 0.02, 95%CI = (0.01 to 0.05), *P* < 0.01) and MCS (β = 0.03, 95%CI = (0.01 to 0.07), *P* < 0.01), via women’s MCS.

Regarding the relation of mothers’ diabetes status and HRQoL with the HRQoL of their children (Table 3: Model 2), mother’s diabetes was not significantly associated with the HRQoL of their children (β = 0.01, *P* = 0.72). However, maternal diabetes had a significant indirect association with children’s HRQoL via mothers’ MCS (β = − 0.02, 95%CI = (− 0.05 to − 0.01), *P* < 0.01). Maternal age (β = − 0.09, *P* = 0.04) and MCS (β = 0.18, *P* = 0.01) were significantly associated with the HRQoL of their children. Although, mother’s education did not have a direct association with children’s HRQoL (β = − 0.01, *P* = 0.74), it was indirectly associated with HRQoL in children via maternal MCS (β = 0.03, 95%CI = (0.01 to 0.06), *P* < 0.01).

## Discussion

Study results show that even after adjusting for potential confounders, diabetes type 2 had a detrimental impact on the mental HRQoL in women but was not directly associated with the HRQoL of their family members. However, these negative associations between diabetes and women's mental HRQoL could affect different aspects of the HRQoL in their spouses and children.

Current findings regarding the negative association between type 2 diabetes and mental HRQoL in women are to some extent consistent with previous findings documented on this association in both mental and physical HRQoL among Iranians and individuals from other countries [31–33]. However other data reveal this association only in the physical HRQoL among older Japanese adults [34]. Existing data shows that women with diabetes feel they are inadequately supported by their family members, friends and health care providers. They also experience significant anxiety and depression in managing their disease and fulfilling their responsibilities in family care [35], which leads to increased risk of poor HRQoL in them. [36]. Previous studies show that compared with men, in dealing with diabetes, women are more likely to use negative coping styles such as emotion-focused and avoidant strategies, which could put them at risk for emotional problems [37]. In this regard the well-documented association between diabetes and sexual dysfunction in women [38–40] could negatively influence individuals’ assessments of their well-being [41].

The current results show the lack of a significant association between diabetes in women and HRQoL of their spouses. Based on our knowledge only one study has investigated the HRQoL in spouses of women with diabetes [42] and found similar results. Most studies have examined the familial impact of chronic diseases, including diabetes, in one of the family members, whether wife, husband or child [18, 19], and their findings indicate significant associations of chronic diseases with family wellbeing, especially the emotional dimension of HRQoL [18, 19]. According to our findings, MCS in women with diabetes was directly associated with both mental and physical HRQoL in their spouses. In relation to this finding, previous studies indicate higher levels of anxiety and depression in spouses of women with diabetes, which can be attributed to the emotional states of their wives [43] and their exacerbated/strenuous caring role in families [44]. Compared to the disease as a primary stressor, the responsibility of taking care of the patient and other family members, as the secondary stressor, plays a major role in causing maladaptation among family members [44]. The emotional distress in women due to their chronic disease reduces the ability of the family members to adapt to their condition and, as a result, other aspects of their spouses’ health are also affected [19]. It is possible that, because of multiple responsibilities, spouses of these patients neglect their own needs and healthy lifestyles, resulting in less physical activity [17], more sleeping problems and poor physical health for them [18].

On the other hand, the current study showed that maternal diabetes affected children’s HRQoL via their mothers’ mental HRQoL, findings which are difficult to compare because of the limited studies available examining the direct and indirect impacts of maternal diabetes on the well-being of their children. However, in agreement with our findings, a research conducted on single parent families, showed no significant differences between different aspects of wellbeing in children of mothers with and without chronic diseases, including diabetes [45]. Regarding the direct effect of maternal mental health on the child’s HRQoL, it seems that the mother’s diabetes per se cannot lead to physical and psychological problems for their children, but the effects of the disease on mothers’ parenting practices and the psychological consequences can result in health problems in children [46]. Mothers with physical health problems usually have high levels of parenting stress, which causes problems for them in meeting their child’s basic needs [46]. Anxiety and depression in mothers and hence poor parenting behaviors can have major negative effects on the functioning, adjustment and quality of life in their children [47]. Also, children of mothers with high levels of psychological distress, have fewer healthy behaviors; furthermore, since their nutrition status is not appropriately monitored, this could also affect their physical health [48].

The main strength of this study is that for the first time, it investigates the direct and indirect associations between women’s diabetes and their personal and familial HRQoL among a large Middle-Eastern population. However, this study has few limitations. It lacks some potentially effective variables such as diabetes duration, different stressors and perceived social support. In addition, the current study has been conducted on urban families in Iran which limits the generalizability of findings; further studies on suburban and rural areas are needed to address this issue.

## Conclusions

Findings of this study showed that diabetes type 2, beyond its negative impact on the mental health status of women, could be associated with HRQoL in their spouses and children via this negative association. Based on the current findings, supporting and empowering women with diabetes to use effective coping skills and manage their disease may result in the improvement of their HRQoL and in turn, in the HRQoL of their families.

## Data Availability

The datasets used in the current study are available from the corresponding author on reasonable request.

## Abbreviations

AGFI: Adjusted Goodness of Fit Index
BMI: Body Mass Index
BP: Bodily Pain
CFI: Comparative Fit Index
DF: Degrees of Freedom
GFI: Goodness of Fit Index
GH: General Health
HRQoL: Health-Related Quality of Life
IFI: Incremental Fit Index
MCS: Mental Component Summary
MH: Mental Health
NFI: Normed Fit Index
PCS: Physical Component Summary
PedsQL: Pediatric Quality of Life Inventory version™ 4.0
PF: Physical Functioning
RE: Role Emotional
RMSEA: Root Mean Square Error of Approximation
RP: Role Physical
SEM: Structural Equation Modeling
SF: Social Functioning
SF-12v2: 12-Item Health Survey version 2
SRMR: Standardized Root Mean Square Residual
TLGS: Tehran Lipid and Glucose Study
VT: Vitality
